# Wunderlich syndrome in a patient on hemodialysis

**DOI:** 10.1590/2175-8239-JBN-2023-0011en

**Published:** 2023-04-17

**Authors:** Maria Inês Roxo, Bruno Pepe, Rita Birne

**Affiliations:** 1 Centro Hospitalar de Lisboa Ocidental, Serviço de Nefrologia, Carnaxide, Portugal.; 2 Universidade NOVA de Lisboa, Lisboa, Portugal.

Dear Editor,

Wunderlich syndrome (WS) is an uncommon but potentially life-threatening entity and consists of spontaneous renal or perirenal hemorrhage^1^.

We report a case of a 56-year-old man with a history of end-stage renal disease due to APOL1-mediated kidney disease on maintenance hemodialysis who was referred to the emergency department with left flank pain, hypotension, nausea, and vomiting of sudden onset during his routine hemodialysis session. He denied other complaints and was not on antiplatelet or anticoagulant drugs. Computed tomography (CT) angiography was performed, revealing 7-centimeter-long kidneys with several cystic lesions, as well as a subacute left kidney hematoma that extended into the perirenal space and pelvic cavity, with no evidence of active bleeding (Figure 1). The patient remained clinically stable; however, there was a hemoglobin drop of 2 g/dL in less than 24 hours. Considering the risks of the procedure and the lack of benefit in preserving the kidney, the patient underwent left nephrectomy. Aside from the need for transfusion support during the surgical intervention, there were no other complications and on the 8^th^ postoperative day he was discharged. The pathological examination of the surgical specimen confirmed acquired cystic kidney disease (ACKD) and identified bleeding in some of the cysts, through the lacerated renal capsule and into the perirenal adipose tissue. There was no evidence of neoplastic disease.

**Figure 1.**
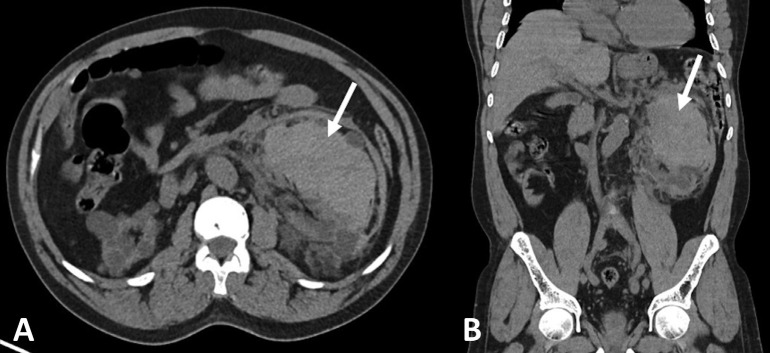
Contrast-enhanced CT showing left renal and perirenal hematoma (white arrows). A – axial view; B – coronal reconstruction.

WS may present with the classic Lenk’s triad, characterized by acute flank pain, palpable mass, and hypotension; nevertheless, atypical presentations are more frequent. Potential underlying etiologies can be varied, and include mostly neoplasms, but also vascular disease, cyst rupture, coagulopathy, or infection^1^. Although cyst hemorrhage is a frequent complication in autosomal dominant polycystic kidney disease (ADPKD)^2^, WS is uncommon both in ADPKD and ACKD^1,2^. In patients on hemodialysis, it appears to result from a combination of factors, namely platelet dysfunction, oral anticoagulant medications, and heparinization of the extracorporeal circuit^3–5^ – nonetheless, WS may be associated solely with cyst or tumor development, without any other predisposing factors^4^. The diagnosis must be confirmed radiologically, ideally by contrast-enhanced CT^1,4,5^. Management may be conservative (with fluid therapy, anticoagulation reversal, analgesics, antibiotics) or interventional (percutaneous embolization or partial/radical nephrectomy)^1^. The first option entails the need for close initial surveillance (in order to intervene in case of deterioration) and subsequent radiological follow-up (so as to monitor the regression of the hematoma and to evaluate for potential underlying neoplasms)^4,5^. While stable patients with benign conditions may be treated conservatively, angiographic embolization might be attempted when active bleeding is detected, and nephrectomy is preferred in the presence of instability or high likelihood of malignancy. Ultimately, the most appropriate treatment should be chosen on an individual basis, depending on clinical stability, evidence of active hemorrhage, suspected cause, and risk/benefit ratio of the available alternatives^1,4^.

## Disclosures

R. Birne reports the following: employed by Diaverum; research funding from Gilead, Bayer, and Boehringer; member in advisory board of Bayer and Boehringer; and member of the speakers bureau of AstraZeneca, Bayer, Boehringer, MSD and Bial. M. Roxo reports the following: employed by Fresenius Medical Care.

## References

[1] Giovini M, Poggiali E, Zocchi P, Bianchi E, Antonucci E, Barbera M (2022). A case of spontaneous renal haemorrhage (wunderlich syndrome) in
an anticoagulated patient.. Eur J Case Rep Intern Med..

[2] Cheng CI, Karvelas NB, Aronowitz P. (2015). Retroperitoneal cyst hemorrhage in polycystic kidney
disease.. Cleve Clin J Med..

[3] Moore AE, Kujubu DA. (2007). Spontaneous retroperitoneal hemorrhage due to acquired cystic
kidney disease.. Hemodial Int..

[4] Ku JH, Kim J, Ha S, Lee JW. (2009). Bilateral spontaneous perirenal haemorrhage in a patient on
haemodialysis.. NDT Plus..

[5] Kawahara T, Kawahara K, Ito H, Yamaguchi S, Mitsuhashi H, Makiyama K (2011). Spontaneous renal hemorrhage in hemodialysis
patients.. Case Rep Nephrol Urol..

